# Exploring invertebrate indicators of ecosystem health by focusing on the flow transitional zones in a large, shallow eutrophic lake

**DOI:** 10.1007/s11356-023-28045-3

**Published:** 2023-06-17

**Authors:** Aimin Hao, Sohei Kobayashi, Fangbo Chen, Zhixiong Yan, Takaaki Torii, Min Zhao, Yasushi Iseri

**Affiliations:** 1 College of Life and Environmental Sciences, Wenzhou University, Wenzhou, Zhejiang 325035 China; 2 National and Local Joint Engineering Research Center of Ecological Treatment Technology for Urban Water Pollution, Wenzhou University, Wenzhou, Zhejiang, China; 3 Zhejiang Provincial Key Laboratory for Water Environment and Marine Biological Resources Protection, Wenzhou University, Wenzhou, 325035 China; 4 Laboratory of Molecular Reproductive Biology, Graduate Division of Nutritional and Environmental Sciences, University of Shizuoka, Shizuoka City, Shizuoka Japan; 5 Institute of Environmental Ecology, Environmental Ecology Division, Idea Consultants Inc., Yaizu City, Shizuoka Japan

**Keywords:** Lakes, Rivers, Eutrophication, Benthic invertebrates, Purification, Transitional zones, Wind-wave actions, Saline species

## Abstract

The river–lake transitional zone provides a unique environment for the biological community and can reduce pollution inputs in lake ecosystems from their catchments. To explore environmental conditions with high purification potential in Lake Taihu and indicator species, we examined the river-to-lake changes in water and sediment quality and benthic invertebrate communities in the transitional zone of four regions. The spatial variations in the environment and invertebrate community observed in this study followed the previously reported patterns in Taihu; the northern and western regions were characterized by higher nutrient concentrations in water, higher heavy metal concentrations in sediment, and higher total invertebrate density and biomass dominated by pollution-tolerant oligochaetes and chironomids. Although nutrient concentrations were low and transparency was high in the eastern region, the taxon richness was the lowest there, which disagreed with the previous findings and might be due to a poor cover of macrophytes in this study. The river-to-lake change was large in the southern region for water quality and the invertebrate community. Water circulation induced by strong wind-wave actions in the lake sites of the southern region is assumed to have promoted photosynthetic and nutrient uptake activities and favored invertebrates that require well-aerated conditions such as polychaetes and burrowing crustaceans. Invertebrates usually adapted to brackish and saline environments are suggested to be indicators of a well-circulated environment with active biogeochemical processes and a less eutrophic state in Taihu, and wind-wave actions are key to maintaining such a community and natural purifying processes.

**Supplementary Information:**

The online version contains supplementary material available at 10.1007/s11356-023-28045-3.

## Introduction

Eutrophication due to nutrient and organic loads from catchments and the deterioration of aquatic ecosystems has been a serious problem in many ponds and lakes worldwide (Dodds et al. 2009; Harper 1992; Smith and Schindler 2009; Shadrin et al. 2018). Cyanobacterial blooms are considered one of the worst outcomes of eutrophication because of their heavy water surface cover and their inedible and toxic nature, which consequently damage various important phyto- and zooplankton, submerged plants, fish, and other aerobic animals (Glibert 2017; Gong and Xie 2001; Helminen et al. 2000; Landsberg et al. 2020; Moustaka-Gouni and Sommer 2020; Rabalais et al. 2010; Yakovenko et al. 2022). Natural and healthy ecosystems (i.e., in good ecological status) are expected to possess indigenous organisms and biogeochemical processes to cope with moderate nutrient and organic loads, known as self-purification (Grizzetti et al. 2019; Jiang and Shen 2006). Knowing the key organisms and habitats involved in such processes is essential for understanding aquatic systems’ potential structure and function and addressing suitable and sustainable restoration.

**Fig. 1:**
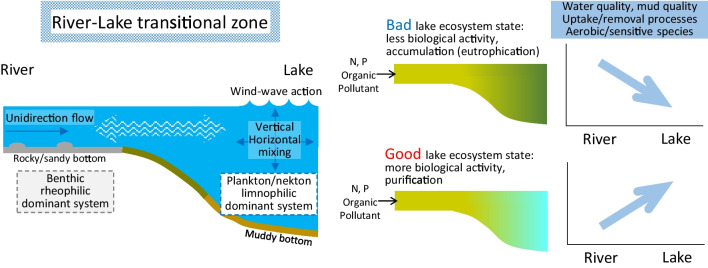
A schematic view of the river–lake transitional zone and expected river-to-lake changes

Rivers from various directions often flow into a lowland lake. A river flow decelerates as it approaches a lake and mixes with the lake water as it enters the lake. The river–lake ecotone or transitional zone includes the river section where the flow is substantially decelerated by the downstream lake and the lake section where the decelerated flow continuously directs to the lake center (Huang et al. 2016; Tian et al. 2020; Wu et al. 2017) (Fig. 1). Sediment and organic matter from upstream are temporarily retained (deposited) in the transitional zone because the flow loses the energy to suspend and transport the particles. Such a temporal retaining nature, a continuous exchange of water, and a mixture of river and lake waters provide a unique environment for the aquatic community (Tian et al. 2020). The transitional zone can also have profound impacts on the lake ecosystem through reducing inputs of nutrients and pollution from the catchments (Wu et al. 2017; Jiang et al. 2019, Yan et al. 2022). The trophic state would be improved from river to lake if nutrients and organic matter are actively removed by the inherent biogeochemical processes in lake ecosystems, while it would be aggravated with flow attenuation or if the biogeochemical processes in the lake have been deteriorated by eutrophication (Fig. 1). Thus, a longitudinal (streamwise) shift in water quality, sediment quality, and biological communities within a transitional zone could reflect the ecological status of the adjacent lake region.

Lake Taihu is a large and shallow lake (the surface area is the third-largest in China) that has been subjected to eutrophication since the 1980s, corresponding to economic growth and severe nutrient loads from industries and urban areas in the catchments. According to many various studies conducted, Taihu is one of the most famous lakes in China and the world in the study of lake eutrophication, especially cyanobacterial blooms (Zhang et al. 2016b; Wang et al. 2022). Several studies of eutrophication in Taihu have focused on benthic invertebrates, which play important roles in the food web and biogeochemical processes and are effective environmental indicators in aquatic systems. A long-term increase in oligochaete worms and chironomid midges and a decrease in bivalve clams and gastropod snails by eutrophication in Taihu have been suggested (Cai et al. 2015, 2012; Peng et al. 2019). The decline and spatial distribution of *Corbicula fluminea* (Asian clams) have received a great concern for the fisheries of Taihu (Han et al. 2019). Among the five large lowland lakes near the Yangtze River, Taihu was characterized by high β-diversity, which suggests high spatial heterogeneity in the invertebrate community and the occurrence of brackish or saline species, including polychaete worms and burrowing crustaceans (Cai et al. 2017b). The spatial distribution of benthic invertebrates is considered to reflect the trophic state of the lake region in Taihu (e.g., Cai et al. 2017a, 2012; Chen et al. 2018a; Xie et al. 2016). The northern region, especially inside bays, was subjected to severe eutrophication (i.e., high phosphorus loads and intense cyanobacterial blooms) and was dominated by oligochaetes and chironomids. In contrast, the east, especially inside bays, had clear water with lower concentrations of nutrients and was rich in aquatic vegetation and more dominated by snails and clams. The open region (lake center, west and south coastal) was characterized by strong wind-wave action inducing flow dynamics (vertical mixing and lateral flow) and was favored by the Asian clams, polychaetes, and certain species of crustaceans (i.e., amphipods and isopods), all of which are brackish, saline, or euryhaline species. Inlet rivers are distributed in all directions in Taihu, while a few outlet rivers are distributed in the eastern half of the lake. Many invertebrate taxa are common in rivers and lakes, and the invertebrate community in rivers varies according to nutrient loads and aquatic vegetation coverage (Zhang et al. 2014). However, few studies have compared invertebrate communities between lakes and rivers, and few studies have examined changes in invertebrate communities in river–lake transitional zones.

To explore the areas and environmental conditions with high purification potential in Lake Taihu and indicator invertebrates, in this study, we examined the river-to-lake changes in water quality, sediment quality, and invertebrate communities in the transitional zone of four regions (north, west, south, and east). We assumed that improvement in water and sediment quality from the river to the lake reflects the purification potential of the area (Fig. 1). Because most invertebrates in lowland rivers and lakes can tolerate conditions of low oxygen availability and organic matter deposition (and thus, usually assigned as pollution-tolerant taxa, Wang and Yan 2004), invertebrates sensitive or tolerant to eutrophication are poorly understood. We explored indicator invertebrates based on their distributions in the transitional zones. We hypothesized that the purification potential is high in the east area according to previous studies showing the high taxonomic richness of benthic invertebrates in the east bays (Cai et al. 2017a, 2012; Chen et al. 2018a; Xie et al. 2016), and the indicator invertebrates would be those abundant in the east.

## Materials and methods

### Study site

**Fig. 2:**
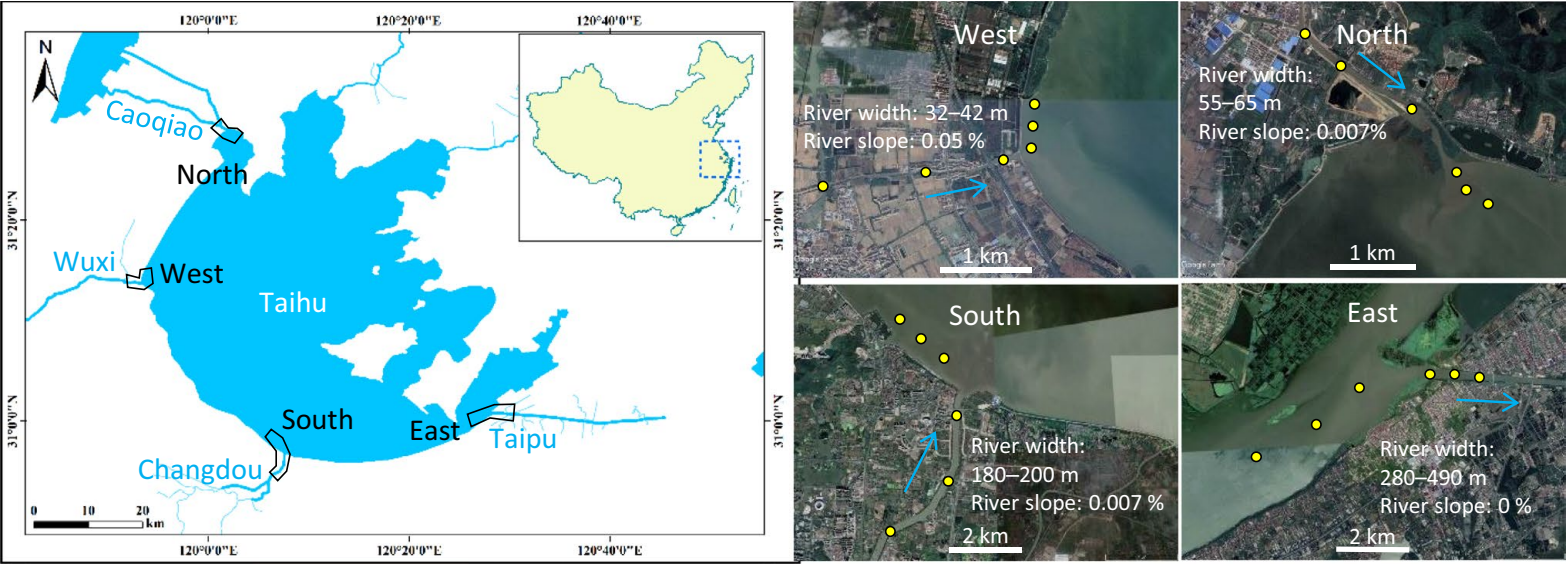
A map of Lake Taihu showing the survey sites in 4 regions (satellite images are from Google Earth). Outlined sections (in the left map) are the spatial range of the survey sites (yellow dots in the right maps). The blue arrow indicates the river flow direction

Lake Taihu (surface area: 2338 km^2^, average water depth: 1.89 m) is located on the coastal plain between the Yangtze River Delta and Qiantang River Estuary (Hangzhou Bay). The catchment of the lake covers 36900 km^2^ including a part of three provinces (Jiangsu, Zhejiang, Anhui) and Shanghai City. The annual rainfall ranged from 1300 to 1800 mm (from 2010 to 2018). There are 22 major rivers, including inlet and outlet rivers. Rivers in the west catchments originate from hills and mountains and have relatively high channel slopes, while those in the other directions originate from the plain or cross-catchment water diversion or are outlet rivers with very small channel slopes. We set up the survey sites in four regions (North Zhushan, West Coastal, South Coastal, East Taihu, Fig. 2). According to the health status report of the Lake 2018 by the Taihu Basin Authority of the Ministry of Water Resources (available at http://www.tba.gov.cn/slbthlyglj/thjkzkbg/content/slth1_09f7d6b21629439f9891c7fd70ad49d8.html, accessed on 20 February 2023), the concentrations of total nitrogen (TN) and total phosphorus (TP) were the highest in the northern region, followed by the western, southern, and eastern regions. The density of cyanobacteria and Chlorophyll-a (Chl-a) concentration in water were the highest in the west region, followed by the north, south, and east regions. Aquatic vegetation (submerged plants) occurred exclusively in the east region according to the report. One of the largest rivers in each region was selected for the survey (north: Caoqiao, west: Wuxi, south: Changdou, east: Taipu). The river in the east is a lake outlet, and the other rivers are lake inlets. For the navigation of large ships, the rivers in the south and east are wider and deeper than those in the north and west (Fig. 2). In each region, three river sites and three lake sites were set up within 3-4 km of the lake inlet (i.e., the river mouth) (a total of 24 sites).

### Field survey

Field surveys at 24 sites were conducted in late April 2021, when the river flow and lake water level were stable and benthic invertebrates were usually more abundant and grew larger before the reproduction season. Lake and river sites in the same region were visited on the same day. At each site, the measurement and sampling were performed at an offshore location (i.e., far from the bank) (Fig. 2). The water quality (i.e., electric conductivity = EC, dissolved oxygen = DO, pH, Chl-a, turbidity, and oxidation-reduction potential = ORP) was measured from the surface to the bottom using a multiparameter water quality meter (Hydrolab DS5X including Hach LDO® sensor; OTT Hydromet GmbH, Kempten, Germany). Water transparency was measured using a Secchi disk. A 500 mL water sample for nutrient analysis was collected from the surface (at a depth of 0.1 m) and bottom (0.5 m above the bottom) using a 3-L Van Dorn water sampler. Four bottom mud samples were collected using a 15-cm Ekman–Birge grab sampler. The sampled mud was homogenized in a bucket, and one-fourth of the volume was resampled for mud analysis. The rest of the mud was washed using a 0.25-mm sieve, and materials including invertebrates on the sieve were collected and preserved with formalin (final concentration: 5%) in a 500-mL bottle. Water and mud samples were transported in a cooler box to the laboratory.

### Laboratory procedures

TN, TP, ammonia (NH_4_-N), nitrite (NO_2_-N), nitrate (NO_3_-N), and phosphate (PO_4_-P) were determined using a Skalar autoanalyzer (San++ continuous flow analyzer, Skalar Analytical B.V., Breda, Netherlands) following Hao et al. (2022). The proportions of TN and TP, apart from inorganic-N and -P, were treated as organic-N and -P, respectively.

Mud pH and ORP were analyzed using portable water quality meters (HQ40D and 2100Q; Hach, Loveland, USA). Moisture was determined by weight loss by drying at 105°C for 24 h. Organic content was evaluated by weight loss on ignition (LOI) at 550°C for 2 h. Silt-clay content (<0.063-mm mesh) was determined by dry weight after drying at 70°C for 48 h and sieving. Acid-volatile sulfide (AVS) was liberated under acidic conditions, and sulfide precipitated with zinc was quantified by iodometry (Pu et al. 2006). The organic content, TN, and TP in the sediment samples were quantified by ultraviolet spectrophotometry after alkaline potassium persulfate digestion following the national standard methods of the alkali fusion Mo-Sb Anti spectrophotometer (GB 9834-88, CJ/T221-2005, HJ 632-2011). Concentrations of four heavy metals (Ni, Cu, Cd, and Pb) were quantified by an inductively coupled plasma mass spectrometer (NexION® 2000, PerkinElmer, Inc., Waltham, MA, USA) after digestion using a concentrated HNO_3_-HCL mixture (Liu et al. 2020).

Invertebrates in samples were gently washed on a 0.5-mm-mesh sieve and separated from other materials in a white tray filled with water. The invertebrates were then temporarily preserved with 75% ethanol. We identified invertebrates to the lowest possible taxonomic level (usually genus) under a stereomicroscope (NEXCOPE NSZ818, Ningbo Yongxin Optical CP., LTD., Ningbo, China) at 10–135× magnification. The taxonomic identification was based on Liu et al. (1979), Morse et al. (1994), and some common keys of related freshwater and brackish invertebrates in Japan. The number of each taxon was counted, divided by the sampled area (0.0675 m^2^), and expressed as density (individuals m^−2^). The wet weight (mg) of each taxon was measured for large invertebrates (e.g., snails and clams) or estimated for smaller invertebrates (e.g., polychaetes, oligochaetes, and insects) using data from Cai et al. (2017a) and expressed as biomass (mg m^−2^). The taxa that occurred in each sample were counted and expressed as taxon richness.

### Data analyses

Differences in water quality, sediment quality, and invertebrate community were tested by two-way analysis of variance (ANOVA) with region (N, W, S, and E) and habitat (lake, river) as factors and site as replication (*n* = 3). Water quality included water temperature, EC, DO, pH, ORP, Chl-a, turbidity, and transparency (Secchi depth) using the mean of vertical 0.1-m interval data and TN, NH_4_-N, NO_2_-N, NO_3_-N, organic-N, TP, PO_4_-P, and organic-P using the mean of surface and bottom data. Sediment quality included soil moisture, organic content (LOI), silt-clay content, AVS, pH, ORP, and concentrations of TN, TP, Ni, Cu, Cd, and Pb. The invertebrate community included the total density, total biomass, taxon richness, and densities of the major groups. Density and biomass data were log(*x*+1)-transformed before the analyses.

Principal component analysis (PCA) using a correlation matrix was performed to summarize the spatial heterogeneity in water and sediment quality. PCA was performed among 24 sites for water and sediment quality separately. The PCA scores were used as the state of water and sediment quality of each site. Differences in mean PCA scores between lake and river were calculated as indices of the direction and degree of river-to-lake change for each region.

Nonmetric multidimensional scaling (NMDS) ordination, which plots communities in 2-dimensional space so that the distances between communities correspond to their dissimilarities, was used to visually examine differences in the community structure among the four regions and between lakes and rivers. A matrix of Bray–Curtis distances was used in the ordination. Permutational multivariate analysis of variance (PERMANOVA) using distance matrices was performed to test the difference in the community structure among the regions and between lake and river statistically. Finally, hierarchical cluster analysis using Ward’s cluster algorithm with Bray–Curtis distance was performed to detect invertebrate taxa relevant to the river-to-lake change.

For all tests, an *α*-value of 0.05 was used to determine the significance of the effects. All statistical tests and multivariate analyses were performed using R software (version 4.0.3; R Development Core Team, Vienna, Austria) with the packages “multcomp,” “MASS,” “vegan,” and “cluster.”

## Results

### Water quality

**Fig. 3:**
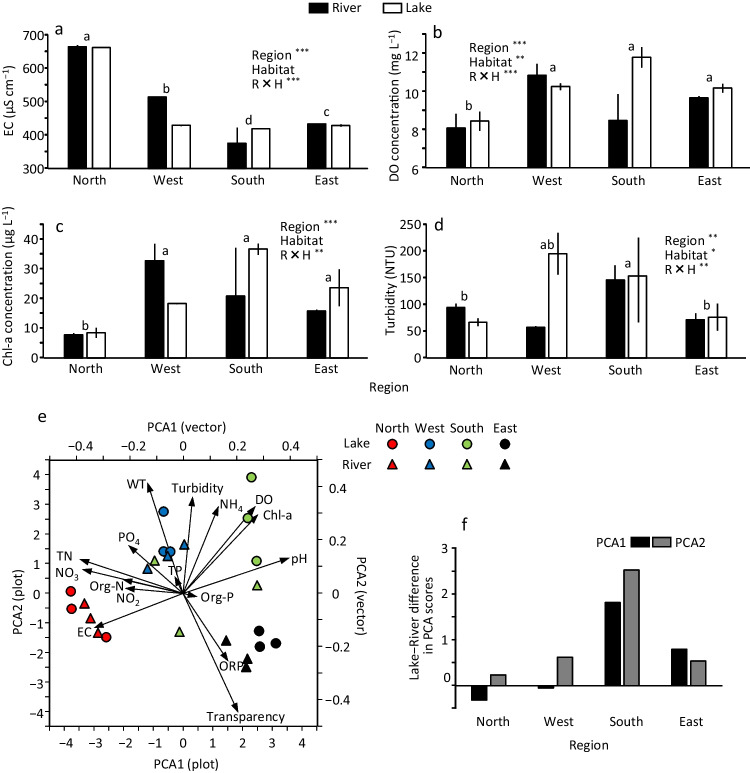
Differences in some water quality variables (**a**: EC, **b**: DO, **c**: Chl-a, **d**: turbidity) among four regions and between lake and river, **(e)** PCA performed among 24 sites for the all-water quality variables, and **(f)** the lake–river difference in mean PCA scores in each region. Significance of the effects of different factors examined by ANOVA is shown by asterisks (^*^p < 0.05, ^**^p < 0.01, and ^***^p < 0.001). Error bars are SD. Different superscripts denote significant difference between the two regions (Tukey’s multiple comparison test). See also Fig. S2 for the difference in other water quality variables among the regions and between lake and river.

There was no large and consistent pattern in the vertical profile of water quality variables at 24 sites (Fig. S1), and thus, the mean of vertical 0.1-m interval data at each site was analyzed to compare among sites, and the mean of the 3 sites with the results of the statistical test is shown in Fig. 3 and Fig. S2. EC (range: 375–666 μS cm^−1^) was the highest in the north, followed by the west, east, and south, and differed significantly among them (Fig. 3a). With a significant interaction (region × habitat) effect, EC decreased approximately 85 μS cm^−1^ from river to lake in the west. DO (range: 8.0–11.8 mg L^−1^) was significantly lower in the north than in the other regions and was also significantly higher in the lake than in the river (Fig. 3b). With a significant interaction effect, the DO increased by approximately 3.3 mg L^−1^ from river to lake in the south. The pH (range: 7.3–8.2) was significantly lower in the north than in the other regions and significantly lower in the west than in the south (Fig. S2). Chl-a (range: 7–37 μg L^−1^) was significantly lower in the north than in the other regions (Fig. 3c). With a significant interaction effect, Chl-a increased approximately 10 μg L^−1^ from the river to the lake in the south. Turbidity (range: 66–196 NTU) was significantly higher in the west and south than east and significantly higher in the river than in the lake (Fig. 3d). Water transparency (range: 12–24 cm) was significantly higher in the east than in the other regions, with the west being the lowest (Fig. S2). TN (range: 1.1–4.6 mg L^−1^) was significantly higher in the north and west than in the south and east (Fig. S2). NO_3_-N (range: 0.4–2.7 mg L^−1^), the dominant N form, showed similar and much conspicuous differences among the regions. Organic-N (range: 0.2–2.0 mg L^−1^) was significantly higher in the river than in the lake. TP (range: 0.03–0.10 mg L^−1^) did not show significant differences among regions or between the river and the lake (Fig. S2). PO_4_-P (range: 0.01–0.03 mg L^−1^) was significantly higher in the north than in the west and east and was significantly higher in the lake than in the river.

The differences in water quality among the four regions and between the river and lake were summarized in PCA (Fig. 3e). The first two components (PCA 1 and 2) accounted for 33.9% and 18.7% (cumulatively 52.7%) of the total variance in the water quality among the 24 sites, respectively. PCA 1 positively correlated with pH, DO, and Chl-a, negatively correlated with TN, NO_3_-N, and EC, and possibly described photosynthetic and nutrient removal potentials. PCA 2 positively correlated with turbidity, water temperature, and NH_4_-N, negatively correlated with transparency and ORP, and possibly describes a water perturbation or organic matter decomposition potential. On the PCA diagram, the four regions were discretely separated, with greater PCA 1 scores for the south and east than for the north and west, and lower PCA 2 scores for the east than for the other regions. The lake and river sites were separated most conspicuously in the south region. The difference in mean PCA scores between the lake and river was calculated and compared (Fig. 3f). The increases in PCA scores from river to lake, as indices of the change in water quality, were the largest in the south and lowest in the north for both PCA 1 and 2.

### Sediment quality

**Fig. 4:**
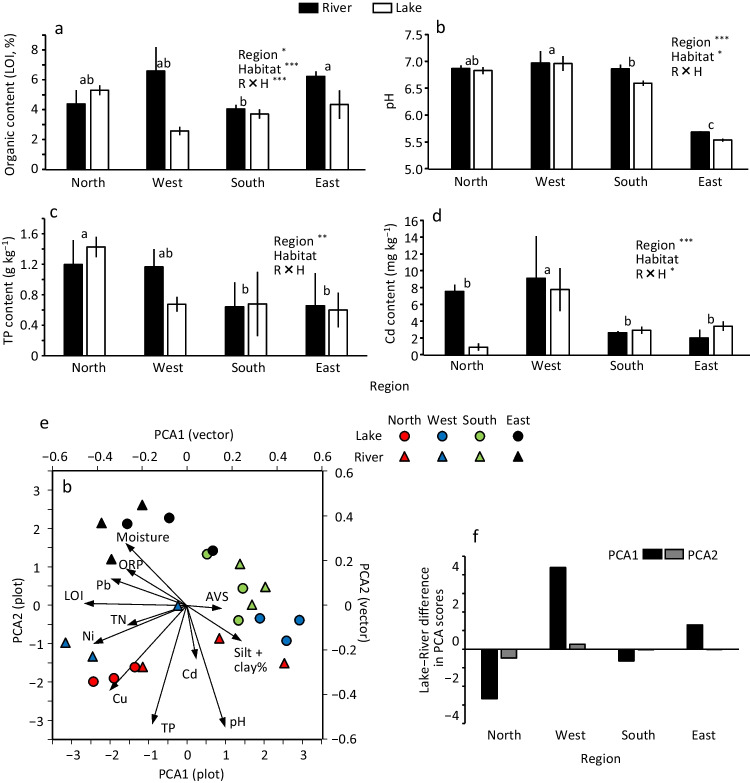
Differences in some sediment quality variables (**a**: LOI, **b**: pH, **c**: TN, **d**: Cd) among four regions and between lake and river, **(e)** PCA performed among 24 sites for the all-sediment quality variables, and **(f)** the lake–river difference in mean PCA scores in each region. Significance of the effects of different factors examined by ANOVA is shown by asterisks (^*^p < 0.05, ^**^p < 0.01, and ^***^p < 0.001). Error bars are SD. Different superscripts denote significant difference between the two regions (Tukey’s multiple comparison test). See also Fig. S3 for the difference in other sediment quality variables among the regions and between lake and river

The differences in sediment quality variables among the four regions and between lake and river are shown in Fig. 4 and Fig. S3. Moisture (range: 34–48%) did not show a significant difference among regions or between the river and lake, while the interaction effect (region × habitat) was significant (Fig. S3); moisture increased 4–5% from river to lake in the north and east and decreased approximately 10% in the west. The organic content (LOI, range: 2.6–6.6%) was significantly higher in the east than in the south and significantly higher in the river than in the lake (Fig. 4a). The interaction effect was significant, and the decrease in organic content from river to lake was most conspicuous in the west (4.0%). The silt-clay content (range: 71–96%) was significantly higher in the lake than in the river (Fig. S3). AVS (range: 13–68 mg kg^−1^) tended to be higher in the river of the south, although no significant difference was observed. The pH (5.5–7.0) was significantly lower in the west than in the other regions and was significantly lower in the lake than in the river (Fig. 4b). With a significant interaction effect, the decrease in TN from the river to the lake was conspicuous in the west and south (Fig. S3). TP was significantly higher in the north than in the south and east (Fig. 4c). Concentrations of Ni (71–103 mg kg^−1^), Cu (27–119 mg kg^−1^), Cd (0.9–9.2 mg kg^−1^), and Pb (23–42 mg kg^−1^) tended to be higher in the north and west than in the south and east (Fig. 4d, Fig. S3). With a significant interaction effect, the decrease in Ni and Cu from the river to the lake was large in the west, while the decrease in Cd was large in the north.

The differences in sediment quality among the four regions and between the river and lake were summarized in PCA (Fig. 4e). The first two components (PCA 1 and 2) accounted for 29.3% and 17.2% (cumulatively 46.6%) of the total variance, respectively. PCA 1 was negatively correlated with the organic content (LOI), and the concentrations of Ni and Pb and possibly describe the reduction in organic matter and heavy metal accumulation. PCA 2 positively correlated with moisture and negatively correlated with pH and TP, and possibly describes mud softness and oxidative potential. The lake in the west and south and the river in the north and south had higher PCA 1 scores than the rest of the sites. The east sites had higher PCA 2 scores than the sites in the other regions. The increase in the PCA 1 score from river to lake was the largest in the west, while the difference in the PCA 2 score was small in all regions (Fig. 4f).

### Invertebrate community

**Fig. 5:**
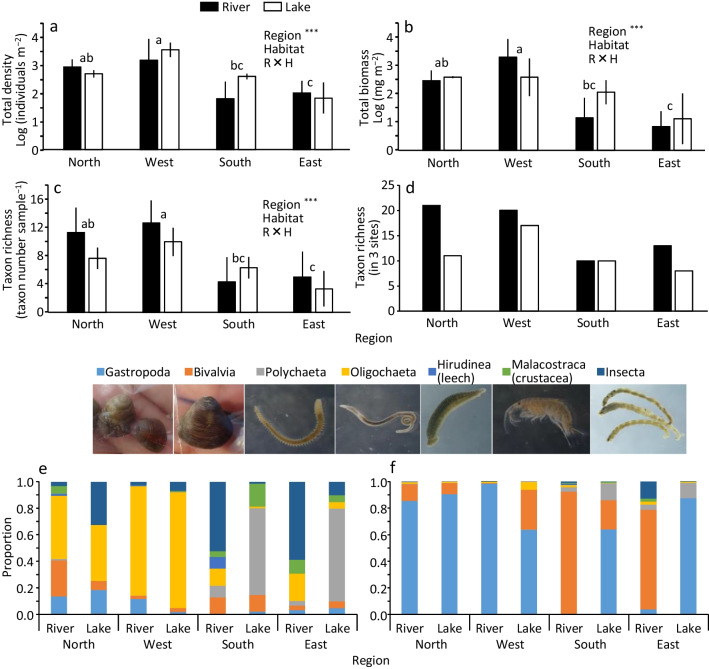
Differences in total density **(a)**, total biomass **(b)**, taxon richness (**c**: per sample, **d**: per 3 sites), and taxonomic composition (e: density, f: biomass) among the four regions and between the lake and river. The significance of the effects of different factors examined by ANOVA is shown by asterisks (^*^p < 0.05, ^**^p < 0.01, and ^***^p < 0.001). Error bars are SD. Different superscripts denote significant differences between the two regions (Tukey’s multiple comparison test)

In total, 43 invertebrate taxa occurred across all sites (Table S1). The total invertebrate density (range: 35–3439 individuals m^−2^), total invertebrate biomass (6–2018 mg wet weight m^−2^), and taxon richness (3.3–12.7 taxa sample^−1^) were significantly higher in the west than in the south and east (Fig. 5a–c). The total density and total biomass were especially low in the south river and the east river and lake. Additionally, taxon richness across 3 sites (8–21 taxa) was also higher in the north and west than in the south and east (Fig. 5d). The increases in total density, total biomass, and taxon richness from river to lake were higher in the south than in the other regions. The dominant invertebrate groups in terms of density were oligochaetes in the north and west, polychaetes in the lakes of the south and east, and insects in the rivers of the south and east (Fig. 5e), while those in terms of biomass were gastropods or bivalves in all regions (Fig. 5f). The mean density of the major groups was compared between the river and lake sites and among regions (Fig. S4). The densities of gastropods, bivalves, and oligochaetes were significantly higher in the north and west than in the south and east. Meanwhile, the density of polychaetes was significantly higher in the south than in the north and east. The interaction effect was significant in the densities of polychaetes and crustaceans, and the increase from river to lake was the greatest in the south. The density of leeches was significantly higher in the river than in the lake.

**Fig. 6:**
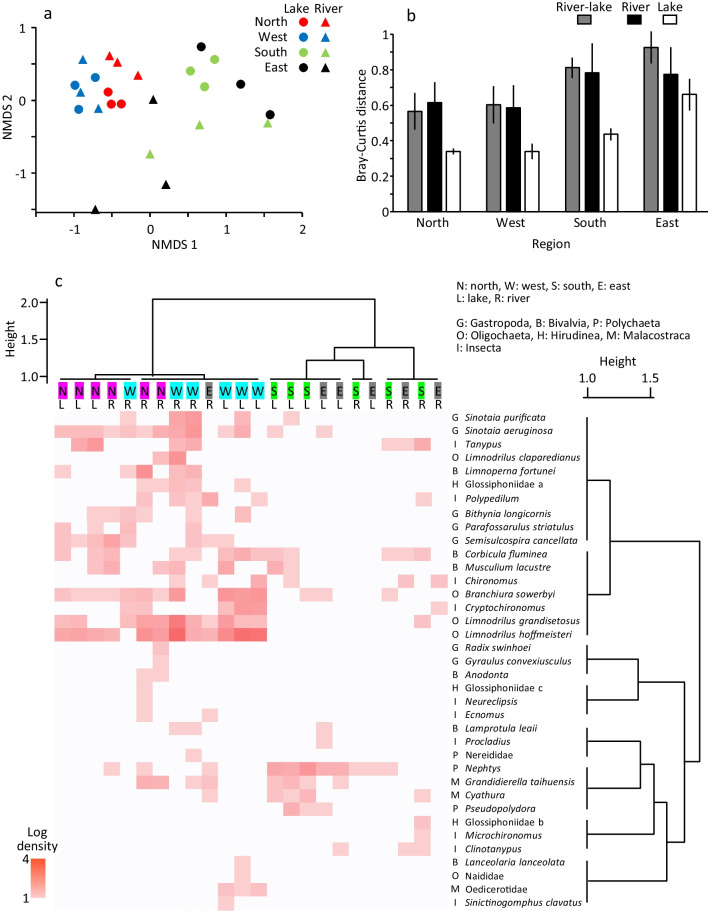
Plots of 24 sites in NMDS ordination of invertebrate community **(a)**, Bray–Curtis community distance in each region **(b)**, and hierarchical cluster analysis by Ward’s clustering algorithm and density heatmap **(c)**. Error bars are SD

Differences in invertebrate community structure among the regions were evident in the NMDS ordination (Fig. 6a). The communities in the north and west were concentrated with negative Axis 1 scores, and they were also separated from each other with lower Axis 1 scores for the west. The lakes in the south and east had positive Axis 1 scores, with higher scores for the latter. Meanwhile, the south and east rivers had lower Axis 2 scores; the south and east rivers varied among sites in Axis 1 and 2 scores, respectively. Such variation in community is due to the small number of taxa (1–4 taxa) in these sites. PERMANOVA suggested that the invertebrate community differed significantly among the 4 regions (*F*-statistic: 3.83, *R*^2^: 0.36, *p* = 0.001) but did not differ significantly between the lake and river across the regions (*F*-statistic: 0.72, *R*^2^: 0.03, *p* = 0.66).

The Bray–Curtis distance between the river and lake ranged from 0.56 to 0.92, and the distance was greater in the south and east than in the north and west (Fig. 6b). In each region, the Bray–Curtis distance between lake sites was relatively low (0.34–0.66), while the distance between river sites (0.58–0.78) was similar to that between river and lake.

In the cluster analysis with Bray–Curtis distance and Ward’s clustering algorithm, invertebrate communities were first partitioned into a north-west group and a south-east group (Fig. 6c). One river site in the east was assigned to the former group. The former group was further partitioned into a group with mainly the north lake sites and a group with mainly the west sites. The south-east group was further partitioned into a group with mainly lake sites and a group with mainly river sites.

Invertebrate taxa were first partitioned into a group that was distributed mainly in the north and west and a group distributed mainly in the south and east (or distributed only in a few sites for the latter) (Fig. 6c). The former group was further partitioned into a group relatively abundant in the north, including gastropods *Sinotaia aeruginosa*, *Semisulcospira cancellata*, and chironomid *Tanypus*, and a group relatively abundant in the west, including oligochaetes *Branchiura sowerbyi*, *Limnodrilus grandisetosus*, *Limnodrilus hoffmeisteri*, and chironomid *Cryptochironomus* (bivalve *C. fluminea* were abundant in the west and south). Under the group distributed in the south and east, the polychaetes *Nephtys*, *Pseudopolydora*, crustaceans *Cyathura*, and *Grandidierella taihuensis*, which were all distributed mainly in the south lake and were relatively abundant, constituted a small group. Meanwhile, many other taxa occurred only in one of the regions (e.g., the bivalve *Lanceolaria lanceolata*, oligochaete Naididae, crustacean Oedicerotidae, and dragonfly *Sinictinogomphus clavatus* occurred only in the west lake, and gastropod *Radix swinhoei* and caddisfly *Neureclipsis* occurred only in the north river).

## Discussion

In this study, we focused on the river–lake transitional zones and examined the differences in the river-to-lake changes in water quality, sediment quality, and benthic invertebrate communities among the 4 regions of Lake Taihu. Contrary to our initial expectation, the changes in water quality and the invertebrate community were large, especially in the south. In the following, possible features and processes that led to the large river-to-lake changes in the south and indicator invertebrates of good lake ecosystems are discussed.

### Potential factors of the river–lake differences

**Fig. 7:**
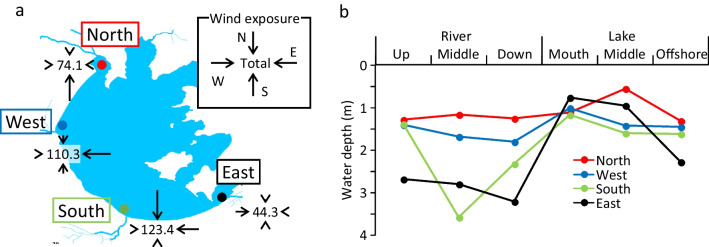
Effective fetch (indicator of wind exposure) calculated for the lake site of 4 regions **(a)** and the water depth profile during the survey in the 4 regions **(b)**

A large change from river to lake was observed in water quality and the invertebrate community in the south. The changes in the water included increases in DO, pH, and Chl-a and decrease in TN (organic fraction), which can indicate increases in photosynthetic, oxygenation, and/or nutrient removal activities. AVS and TN in sediment also tended to decrease from river to lake in the south. The changes in the invertebrate community included the increase in polychaetes (*Nephtys*, *Pseudopolydora*) and burrowing crustaceans (*Cyathura*, *G. taihuensis*), which are a characteristic group of open areas in the middle of Taihu (Cai et al. 2017a, 2012, 2011). According to our following surveys in the south, the difference in the invertebrate community between the river and lake was consistent throughout the year (Fig. S5). The changes in water quality may suggest that algal activities in the water column are promoted from river to lake by the cessation of unidirectional flow and deposition of suspended organic matter. The south coast is one of the regions exposed to strong wind-wave action in Taihu (Cai et al. 2017a). Wind exposure and direction are considered important determinants of the spatial distribution of invertebrate communities in various shallow lakes and coasts through affecting vertical mixing and oxygen availability at the bottom, resuspension of sediment and turbidity of water, organic matter content in sediment, frequency of stochastic disturbance, and dispersion and colonization of invertebrates (Shanks et al. 2000; Cooper et al. 2014; Deng et al. 2018; Heling et al. 2018; Anufriieva et al. 2022). Following these previous studies, effective fetch, as an indicator of wind exposure (Cai et al. 2016; Marques and Andrade 2017), was simply calculated for lake sites in each region based on the frequency and mean speed of wind from and the distance to shore of 4 directions (i.e., N, E, S, W) (using 2010–2019 wind data, Fig. 7a). Indeed, according to prevailing winds from the north and east in terms of wind speed and frequency, respectively, the effective fetch was the highest in the south, followed by the west. In the south lake, despite the deposition of organic matter that can consume oxygen, the sediment can be sufficiently oxygenated by the vertical mixing of water induced by wind-wave action and may be favored by polychaetes and crustaceans, which burrow into the sediment and consume detritus. The excretion of ammonium (NH_4_) and phosphate (PO_4_) by benthic invertebrates has been suggested as an important nutrient source in Taihu (Ji et al. 2015; Peng et al. 2020). Thus, the greatest increase in the concentrations of NH_4_-N and PO_4_-P from river to lake in the south may be associated with the feeding activities of such invertebrates. The changes in sediment, including the decrease in organic content and heavy metal concentrations, were greatest in the west, which had the second largest wind exposure, although the change in the invertebrate community was small there.

The large change in the invertebrate community in the south may also be associated with peculiarities in the geomorphic condition of the river. The abundance (especially biomass) and taxon richness of invertebrates in river sites were substantially lower in the south and east than in the north and west. According to the topography of the basin, the rivers were steeper in the north and west (0.007–0.05%) than in the south and east (<0.007%), which can lead to greater flow velocities and supply of oxygen for sedentary invertebrates in the former rivers. In addition, the rivers in the south and east were deeper because of the dredging to maintain the navigation of large ships, and the depth was actually unnaturally deeper in the river than in lake sites (Fig. 7b). Flow usually passes through the surface layer of the water column, and due to the increased water residence time and biological oxygen consumption by deposited organic matter in the deep layer, the availability of oxygen for benthic invertebrates may be limited at the bottom (Carter et al. 2021; Thompson et al. 2021). Adverse effects of such deepened channels on water and sediment quality were observed for limited parameters in this study (e.g., lower DO in water and higher AVS in sediment in the south river). Alternatively, hydraulic disturbances forced by frequently passing ships might have impacted invertebrates.

Our results indicate that the ecosystem health and purification potential are good/high in the south. While the eastern lake region had been characterized by clear water, abundant macrophyte, and high species diversity of invertebrates (e.g., Cai et al. 2012, 2011), few studies have paid attention to the ecosystem status of the southern region. Meanwhile, the biomass of Asian clam, an important filter feeder purifying water, is potentially high in the southwestern coastal zone (Han et al. 2019). The invertebrate community in the south lake in this study is similar to the open (pelagic) area in previous studies (Cai et al. 2017a, 2012, 2011), where wind-wave action was strong and Asian clams and polychaetes were abundant. The wind-wave action has been suggested to weaken the role of eutrophication in other shallow lakes (Cai et al. 2016), and the long-term decrease in wind speed in Taihu has been suggested to have enhanced low bottom DO, nutrient release from the sediment, and algal bloom in the northern bay (Deng et al. 2018).

### Indicator invertebrates of natural and healthy lake environments

Polychaetes (e.g., *Nephtys* and *Pseudopolydra*) and burrowing crustaceans (e.g., the amphipod *G. taihuensis* and isopod *Cyathura*), which are usually adapted to saline environments, may be an indicator of natural and healthy environments in lowland lakes. These invertebrates may have adapted evolutionarily to freshwater environments in coastal lakes that were originally saline or brackish environments. Lake Taihu was an estuary or salt marsh in the early and middle Holocene, the freshwater environment was stabilized, and the present saucer-like depression was formed in the middle to late Holocene (after 6000–5000 B.P., Chen et al. 2018b; Wang et al. 2001). Although the water diversion project from the Yangtze River to Taihu since 2002 might have increased the biological connection between the estuary and Taihu (Zhang et al. 2022), these saline invertebrates are considered native based on the records before the project (Cai et al. 2015, 2012). Freshwater-adapted populations of an originally anadromous anchovy (*Coilia nanus*) in Taihu (recently) and the other lakes (in the Pleistocene) in the lower Yangtze River have been shown (Cheng et al. 2019; Xue et al. 2020; Zong et al. 2021). Genetic studies are needed to understand whether these polychaete and crustacean populations in Taihu genetically diverged from or still have gene flow connections with saline populations in the Yangtze River estuary.

Polychaetes and burrowing crustaceans are likely to be less tolerant to pollution and eutrophication than oligochaetes and chironomids in Taihu. Our study and previous studies suggest that polychaetes and crustaceans are distributed mainly in places with well-circulated water by wind-wave actions (Cai et al. 2017a, 2012, 2011). In addition, these invertebrates are distributed vertically near the surface in sediment compared to oligochaetes (e.g., *L. hoffmeisteri*) and chironomids (e.g., *Chironomus*) (Chen et al. 2018a), indicating their requirement for more aerated conditions. A decrease in polychaetes and other saline species and an increase in oligochaetes with eutrophication without a significant change in salinity were observed in some estuaries and brackish lakes (e.g., Brauko et al. 2020; Schückel and Kröncke 2013). Indeed, some oligochaetes, including *L. hoffmeisteri* and chironomid species, are often distributed in low saline and eutrophic environments (Rodriguez et al. 2006; Seys et al. 1999; Wolf et al. 2009). Sediment in eutrophic systems may be acidic by the decomposition of accumulated organic matter and less favored by many saline species that require Ca for their exoskeleton (e.g., shell and chitin) in neutral and basic conditions. In addition, some brackish and saline species benefit from groundwater discharge that continuously refreshes water and supplies nutrients for primary production (Leitão et al. 2015; Silva et al. 2012). Thus, the dominance of polychaetes, burrowing crustaceans, and other invertebrates related to brackish or saline aquatic habitats (e.g., Asian clam) in the benthic invertebrate community is assumed to indicate a well-circulated and less eutrophic environment. The occurrence of brackish/saline species in a freshwater environment is not specific to Taihu but is also true for other lakes located near the sea, and freshwater species can occur in a brackish/saline environment with eutrophication (Brauko et al. 2020; Rodriguez et al. 2006; Seys et al. 1999; Schückel and Kröncke 2013; Wolf et al. 2009). Accordingly, examining the dominance of freshwater and saline species as an indicator of eutrophication may be also useful in other freshwater habitats near the sea, where most freshwater species are pollution-tolerant and unavailable as indicators, and in the brackish/saline habitats with freshwater inputs (e.g., tidal reaches in a river, estuary, or seashore with groundwater inputs).

### Spatial differences in the invertebrate community in Lake Taihu

Some important river-to-lake changes may remain concealed because we conducted the survey only once except for the south, in which the rive-to-lake changes were consistent throughout the year (Fig. S5). Although the difference was smaller than in the south region, there was a certain difference in the invertebrate community between the river and lake in the north region according to NMDS (Fig. 6a). In addition, several invertebrate taxa, including a saline-adapted burrowing crustacean Oedicerotidae, occurred only at the lake sites in the west region. Further studies are needed to clarify the river-to-lake difference in these regions. Nevertheless, the spatial distribution of invertebrates among regions found in this study almost followed that reported in previous studies as in the following.

Our results of the differences in water quality, sediment quality, and invertebrate community were in overall accordance with previous studies in Taihu. EC was the highest in the north, followed by the west, and TN concentrations were higher in the north and west than in the south and east. The northern and western regions, especially northern bays (Meiliang, Zhushan), have often been shown to contain high concentrations of nitrogen and phosphorus from domestic and agricultural areas (e.g., Cai et al. 2017b; Li et al. 2022) and consequently have been subjected to severe occurrence of cyanobacterial blooms every year (Jia et al. 2019; Li et al. 2022; Shi et al. 2017). The tendency of higher concentrations of heavy metals (Ni, Cu, Cd) in the sediment of the north and west in this study also agrees with previous studies that showed higher levels of heavy metals and their toxicity in the northern bays in Taihu (Niu et al. 2015; Zhang et al. 2017). In this study, the total invertebrate density, total biomass, taxon richness, and densities of gastropods and oligochaetes were higher in the north and west than in the south and east. Invertebrate densities are usually high for pollution-tolerant species such as oligochaete *L. hoffmeisteri* and chironomids (and gastropod *Sinotaia aeruginosa*) in the northern region (Cai et al. 2012, 2011; Chen et al. 2018a). In addition, the Asian clam (*C. fluminea*), which demands high DO levels for its life (Han et al. 2019), was the most abundant, and the taxon richness was the highest in the west. Thus, although the north and west in this study were still highly eutrophic, hypoxia that eliminates most benthic invertebrates from the sediment is unlikely to occur, and the rich supply of nutrients contributed to support high abundance and diversity by pollution-tolerant invertebrates.

Although nutrient concentrations were relatively low in the east, sediment tended to be organic-rich and acidic, and benthic invertebrates were poor. In this study, nutrient concentrations were lower and transparency was higher in the east. The eastern region, especially inside the bay, often showed the lowest nutrient concentrations, lowest occurrence of cyanobacterial blooms, highest transparency, and lowest trophic level (Cai et al. 2017a; Jia et al. 2019; Li et al. 2022; Shi et al. 2017). This is possibly due to less nutrient and organic loads from the catchment and the effects of aquatic vegetation, which was most abundant in this region (Cai et al. 2017a, 2011; Liang et al. 2017) and can limit the resuspension of nutrients and sediment (Zhu et al. 2015). The relatively higher moisture, silt-clay and organic content of sediment in the east lake also agree with the description of sediment characteristics in previous studies (i.e., muddy and detritus rich, Cai et al. 2012, 2011). Meanwhile, the lowest taxon richness of invertebrates in the east in this study disagrees with previous studies (Cai et al. 2012, 2011), in which the eastern bays presented the highest invertebrate diversity and evenness and were mainly characterized by gastropods relying on macrophytes for their habitats. Macrophyte cover in the eastern bays and for the whole of Taihu has been decreasing since 2010 (Wang et al., 2019; Zhang et al. 2016a), and transparency has been decreasing correspondingly (Dong et al. 2022; Zhang et al. 2018). Indeed, we did not observe macrophyte cover, the transparency was low in our survey, and gastropods were few in the east. Thus, the low taxon richness of invertebrates in the east may indicate a loss of their important habitats.

## Conclusion

By focusing on the river–lake transitional zones, we revealed the river-to-lake changes in water quality, sediment quality, and benthic invertebrate communities in four regions of Lake Taihu. Our results partially agreed with previous studies on the spatial variations among different regions in Taihu; the northern and western regions were characterized by higher nutrient concentrations in water, higher heavy metal concentrations in sediment, and higher total invertebrate density and biomass dominated by pollution-tolerant taxa such as oligochaetes and chironomids. Although nutrient concentrations were low and transparency was high in the eastern region, the taxon richness was the lowest there, which disagreed with previous studies and might be due to poor macrophyte cover. The river-to-lake change was large in the southern region for water quality and the invertebrate community. Water circulation induced by the strong wind-wave actions in the south lake is assumed to have promoted photosynthetic and nutrient uptake activities and favored invertebrates that require well-aerated conditions, such as polychaetes and burrowing crustaceans. Polychaetes, burrowing crustaceans, and other invertebrates related to brackish or saline habitats are suggested to be indicators of a well-circulated environment with active biogeochemical processes and a less eutrophic state in Taihu, and wind-wave actions are key to maintaining such a community and purifying processes.

## Supplementary information

ESM 1 (DOCX 1.55 mb)

## Data Availability

The datasets generated during this study are not publicly available but are available from the corresponding author upon reasonable request.
