# Supplementary material for: Exploring invertebrate indicators of ecosystem health by focusing on the flow transitional zones in a large, shallow eutrophic lake
Source: Environ Sci Pollut Res Int. 2023 Jun 17;30(34):82717–31. doi: 10.1007/s11356-023-28045-3 (PMC10349724; doi:10.1007/s11356-023-28045-3)
Supplement: Supplementary file 1 — (DOCX 1.55 mb) [file 11356_2023_28045_MOESM1_ESM.docx]

**Figure S1** Vertical profiles of representative water quality variables at the 24 survey sites.

**Figure S2** Differences in water quality variables (not shown in Fig. 3) among four regions and between lake and river. Significance of the effects of different factors examined by ANOVA is shown by asterisks (^*^: *p* < 0.05, ^**^: *p* < 0.01, ^***^: *p* < 0.001). Error bars are SD. Different superscripts denote significant difference between the two regions (Tukey’s multiple comparison test).

**Figure S3** Differences in sediment quality variables (not shown in Fig. 4) among four regions and between lake and river. Significance of the effects of different factors examined by ANOVA is shown by asterisks (^*^: *p* < 0.05, ^**^: *p* < 0.01, ^***^: *p* < 0.001). Error bars are SD. Different superscripts denote significant difference between the two regions (Tukey’s multiple comparison test).

**Figure S4** Differences in density of major invertebrate groups among four regions and between lake and river. Significance of the effects of different factors examined by ANOVA is shown by asterisks (^*^: *p* < 0.05, ^**^: *p* < 0.01, ^***^: *p* < 0.001). Error bars are SD. Different superscripts denote significant difference between the two regions (Tukey’s multiple comparison test).

**Figure S5** Seasonal change in total invertebrate density, taxon richness, and densities of major invertebrate groups in the lake and river sites of the south region. Error bars are SD.

**Table S1** Invertebrate taxa occurrence and mean density (log_10_(number per 0.0675m^2^ +1)). The values higher than the mean are shown in bold.

|  |  | Region | | | |  | Habitat | |
| --- | --- | --- | --- | --- | --- | --- | --- | --- |
| Family/Subfamily | Genus/species | N | W | S | E |  | Lake | River |
| Viviparidae | *Sinotaia purificata* | 0.00 | **0.57** | 0.05 | 0.00 |  | 0.08 | **0.23** |
| Viviparidae | *Sinotaia aeruginosa* | **0.54** | **0.69** | 0.05 | 0.05 |  | 0.30 | **0.37** |
| Bithyniidae | *Bithynia longicornis* | **0.15** | **0.23** | 0.00 | 0.00 |  | 0.08 | **0.12** |
| Bithyniidae | *Parafossarulus striatulus* | **0.13** | **0.18** | 0.00 | 0.00 |  | 0.06 | **0.09** |
| Semisulcospiridae | *Semisulcospira cancellata* | **0.43** | **0.21** | 0.00 | 0.05 |  | 0.14 | **0.20** |
| Lymnaeidae | *Radix swinhoei* | **0.08** | 0.00 | 0.00 | 0.00 |  | 0.00 | **0.04** |
| Planorbidea | *Gyraulus convexiusculus* | **0.05** | 0.00 | 0.00 | 0.00 |  | 0.00 | **0.03** |
| Mytilidae | *Limnoperna fortunei* | **0.32** | **0.28** | 0.00 | 0.00 |  | 0.03 | **0.27** |
| Unionidae | *Lamprotula leaii* | 0.00 | **0.10** | 0.00 | **0.05** |  | 0.03 | **0.05** |
| Unionidae | *Anodonta* | **0.10** | 0.00 | 0.00 | 0.00 |  | 0.00 | **0.05** |
| Unionidae | *Lanceolaria lanceolata* | 0.00 | **0.05** | 0.00 | 0.00 |  | **0.03** | 0.00 |
| Cyrenidae | *Corbicula fluminea* | **0.28** | **0.43** | **0.26** | 0.05 |  | **0.31** | 0.20 |
| Sphaeriidae | *Musculium lacustre* | **0.30** | **0.29** | 0.19 | 0.00 |  | **0.22** | 0.17 |
| Nephtyidae | *Nephtys* | 0.10 | 0.00 | **0.68** | **0.38** |  | **0.46** | 0.13 |
| Spionidae | *Pseudopolydora* | 0.00 | 0.00 | **0.19** | 0.05 |  | **0.12** | 0.00 |
| Nereididae |  | 0.00 | **0.05** | 0.00 | 0.00 |  | 0.00 | **0.03** |
| Branchiurinae | *Branchiura sowerbyi* | **0.40** | **1.04** | 0.10 | 0.05 |  | **0.49** | 0.30 |
| Tubificinae | *Limnodrilus grandisetosus* | **0.60** | **1.02** | 0.08 | 0.05 |  | 0.32 | **0.56** |
| Tubificinae | *Limnodrilus hoffmeisteri* | **1.13** | **1.67** | 0.00 | 0.13 |  | 0.81 | **0.66** |
| Tubificinae | *Limnodrilus claparedianus* | **0.10** | **0.25** | 0.00 | 0.00 |  | 0.00 | **0.17** |
| Naididae |  | 0.00 | **0.05** | 0.00 | 0.00 |  | **0.03** | 0.00 |
| Glossiphoniidae | a | **0.10** | **0.23** | 0.00 | 0.00 |  | 0.03 | **0.14** |
| Glossiphoniidae | b | 0.00 | 0.00 | **0.08** | 0.00 |  | 0.00 | **0.04** |
| Glossiphoniidae | c | **0.05** | 0.00 | 0.00 | 0.00 |  | 0.00 | **0.03** |
| Corophiidae | *Grandidierella taihuensis* | **0.27** | 0.05 | **0.25** | 0.13 |  | 0.15 | **0.20** |
| Oedicerotidae |  | 0.00 | **0.21** | 0.00 | 0.00 |  | **0.10** | 0.00 |
| Anthuridae | *Cyathura* | 0.00 | 0.00 | **0.30** | 0.05 |  | **0.12** | 0.05 |
| Gomphidae | *Sinictinogomphus clavatus* | 0.00 | **0.05** | 0.00 | 0.00 |  | **0.03** | 0.00 |
| Polycentropodidae | *Neureclipsis* | **0.05** | 0.00 | 0.00 | 0.00 |  | 0.00 | **0.03** |
| Ecnomidae | *Ecnomus* | **0.05** | 0.00 | 0.00 | **0.05** |  | 0.00 | **0.05** |
| Chironominae | *Chironomus* | 0.00 | **0.20** | 0.05 | **0.21** |  | 0.10 | **0.13** |
| Chironominae | *Cryptochironomus* | 0.08 | **0.62** | 0.00 | 0.05 |  | **0.27** | 0.10 |
| Chironominae | *Microchironomus* | 0.00 | 0.00 | **0.05** | 0.00 |  | 0.00 | **0.03** |
| Chironominae | *Polypedilum* | 0.08 | **0.18** | 0.05 | **0.16** |  | 0.03 | **0.21** |
| Tanypodinae | *Clinotanypus* | 0.00 | 0.00 | **0.05** | **0.10** |  | 0.03 | **0.05** |
| Tanypodinae | *Procladius* | 0.00 | 0.00 | 0.00 | **0.05** |  | **0.03** | 0.00 |
| Tanypodinae | *Tanypus* | **0.40** | **0.27** | 0.21 | 0.05 |  | 0.20 | **0.26** |
